# Translation, Cross-Cultural Adaptation, and Psychometric Evaluation of the European Portuguese Exercise Benefits/Barriers Scale in Higher Education Students

**DOI:** 10.3390/healthcare14172849

**Published:** 2026-09-04

**Authors:** Jéssica Cunha da Silva, Ricardo Maia Ferreira, Luís Cavalheiro, Pedro Lopes Ferreira, Rui Soles Gonçalves

**Affiliations:** 1 Coimbra Health School, Polytechnic University of Coimbra, Rua 5 de Outubro, 3045-043 Coimbra, Portugal; a2023118180@estesc.ipc.pt (J.C.d.S.); rferreira@ipmaia.pt (R.M.F.); luisc@estesc.ipc.pt (L.C.); 2 Social Sciences, Education and Sport Department, Polytechnic Institute of Maia, N2i, 4475-690 Maia, Portugal; 3 Sport Physical Activity and Health Research & Innovation Center (SPRINT), 4960-320 Melgaço, Portugal; 4 Centre for Health Studies and Research, University of Coimbra (CEISUC), 3004-512 Coimbra, Portugal; pedrof@fe.uc.pt; 5 H&TRC-Health & Technology Research Center, Coimbra Health School, Polytechnic University of Coimbra, Rua 5 de Outubro, 3045-043 Coimbra, Portugal; 6 Centre for Innovative Biomedicine and Biotechnology (CiBB), 3004-512 Coimbra, Portugal

**Keywords:** Exercise Benefits/Barriers Scale, EBBS, exercise, perceived benefits, perceived barriers, cross-cultural adaptation, psychometric validation, higher education students, European Portuguese

## Abstract

Background/Objectives: The Exercise Benefits/Barriers Scale (EBBS) assesses perceived benefits and barriers to exercise, but no European Portuguese version had been psychometrically evaluated. This study aimed to translate, cross-culturally adapt, and evaluate the measurement properties of the EBBS in higher education students. Methods: A methodological cross-sectional study included translation, cross-cultural adaptation, content validity assessment, and psychometric evaluation in 324 students; 72 completed a seven-day retest. Structural validity, internal consistency, corrected item–total correlations, test–retest reliability, measurement error, floor and ceiling effects, and construct validity were examined. Results: The adaptation process supported content validity. The second-order confirmatory factor analysis yielded χ^2^/df = 2.512, CFI = 0.808, TLI = 0.797, RMSEA = 0.068, and SRMR = 0.0774, providing only partial and limited support for the original 43-item factorial structure. Cronbach’s alpha was 0.944 for Benefits, 0.831 for Barriers, and 0.926 for the total score. Test–retest reliability was acceptable (ICC = 0.784–0.847), and no problematic floor or ceiling effects were observed. Construct validity evidence was mixed for exercise motivation but more consistent for physical activity, sedentary behaviour, and exercise participation. Conclusions: The European Portuguese EBBS is culturally appropriate and shows favourable reliability, interpretability, and evidence for several aspects of construct validity. However, the original factorial structure is not adequately confirmed. Generalisability is limited by the single-institution convenience sample, predominantly comprising female and health-school students. Further evaluation in larger, more diverse samples is required.

## 1. Introduction

Physical activity and exercise are closely related but conceptually distinct constructs. Physical activity refers to any bodily movement produced by skeletal muscles that results in energy expenditure, whereas exercise is a planned, structured, and repetitive form of physical activity performed with the purpose of improving or maintaining physical fitness [1,2]. Despite the well-established benefits of regular physical activity and exercise for physical and mental health, insufficient physical activity remains a major public health concern worldwide and is associated with an increased risk of non-communicable diseases and premature mortality [3]. This challenge is particularly relevant in young adults, including higher education students, for whom the transition to academic life is often accompanied by changes in routines, autonomy, time management, and health-related behaviours [4,5].

Higher education students represent a population of particular interest for exercise promotion because this life stage may contribute to the consolidation of behavioural patterns with long-term implications for health. Regular participation among students may be constrained by academic workload, competing priorities, perceived lack of time, low motivation, fatigue, and limited access to suitable environments or opportunities for practice [4,5]. Portuguese evidence also suggests that sedentary behaviour and insufficient exercise remain relevant concerns among higher education students, with potential implications for perceived health and well-being [6]. Therefore, understanding exercise participation in this population requires more than measuring behavioural indicators alone; it also requires assessing the cognitive and affective perceptions that may facilitate or inhibit engagement in structured exercise.

Among the theoretical frameworks used to explain health-promoting behaviours, Pender’s Health Promotion Model provides a particularly relevant basis for examining exercise-related decision-making [7]. The model proposes that health behaviour is influenced by individual characteristics and experiences, behaviour-specific cognitions and affect, and behavioural outcomes, with perceived benefits and perceived barriers occupying a central role in the motivational process [8]. Perceived benefits refer to anticipated positive outcomes that may encourage action, whereas perceived barriers represent anticipated obstacles or costs that may reduce the likelihood of initiating or maintaining a behaviour. In exercise contexts, these perceptions may shape whether individuals interpret exercise as worthwhile, feasible, and personally meaningful, or as time-consuming, effortful, inconvenient, or socially discouraging [9,10].

The Exercise Benefits/Barriers Scale (EBBS) was developed to assess these two complementary perceptual dimensions in relation to exercise [10]. The original instrument comprises 43 items organised into a Benefits Subscale and a Barriers Subscale, with a second-order structure reflecting the broader latent dimensions of perceived benefits and perceived barriers. The EBBS has been used in different populations and has been translated or validated in several cultural and linguistic contexts, including university students, older adults, and clinical or community samples [11,12,13,14,15]. However, the available evidence also indicates that the factorial structure of the EBBS may vary across populations and cultural contexts, reinforcing the need for local validation rather than assuming direct transferability of the original structure [11,12].

This issue is especially important because perceived benefits and barriers are culturally embedded constructs. The meaning attributed to exercise, the social acceptability of exercise behaviours, perceived time constraints, environmental access, family or peer influence, and interpretations of health-related terminology may differ across countries, educational settings, and demographic groups. Consequently, the cross-cultural adaptation of a self-report measure must go beyond literal translation and should ensure semantic, conceptual, cultural, and measurement equivalence [16,17]. Contemporary recommendations for self-report health measurement instruments further emphasise that content validity, structural validity, internal consistency, reliability, measurement error, interpretability, and construct validity should be evaluated when an instrument is adapted for use in a new context [18,19,20].

Although a Portuguese-language version of the EBBS has been developed in another Lusophone context, namely Brazilian Portuguese [15], this version cannot be assumed to be linguistically or culturally equivalent to European Portuguese. Differences in vocabulary, idiomatic usage, educational context, and health-related terminology may affect item interpretation and response behaviour. To our knowledge, no European Portuguese version of the EBBS has been fully translated, cross-culturally adapted, and psychometrically evaluated for higher education students in Portugal. This constitutes a relevant gap, given the need for valid instruments capable of identifying perceived facilitators and barriers to exercise in a population in which health behaviours are still being shaped and may be responsive to institutional and health-promotion interventions.

Therefore, the present study aimed to translate and cross-culturally adapt the EBBS into European Portuguese and to evaluate its psychometric properties in higher education students. Specifically, the study assessed content validity, structural validity, internal consistency, corrected item–total correlations, test–retest reliability, measurement error, interpretability through floor and ceiling effects, and construct validity through predefined hypotheses involving exercise motivation, self-reported physical activity and sedentary behaviour, and known-group comparisons based on current exercise participation.

## 2. Materials and Methods

This methodological cross-sectional study was conducted in two phases: translation, cross-cultural adaptation and content validity assessment of the EBBS, followed by evaluation of the psychometric properties of the adapted version. Permission to adapt the EBBS was obtained from one of the authors of the original scale. The study was approved by the Ethics Committee of the Polytechnic Institute of Coimbra, Portugal, on 28 January 2026 (approval code: 197/2025), and was conducted in accordance with the Declaration of Helsinki [21] and the Oviedo Convention [22].

### 2.1. Translation, Cross-Cultural Adaptation and Content Validity

The translation and cross-cultural adaptation process followed international recommendations for the adaptation of self-report instruments [16,17]. Two independent forward translations from English into European Portuguese were performed by native Portuguese-speaking professional translators fluent in English. The two translations were then synthesised by a multidisciplinary consensus panel with experience in health measurement, physiotherapy/human movement sciences, and cross-cultural adaptation of health-related instruments.

The synthesis process was conducted item by item, considering semantic equivalence, cultural adequacy for the Portuguese context, and the accessibility of the wording for the target population. A back-translation was subsequently performed by an independent native English-speaking translator fluent in Portuguese and blinded to the original version. The back-translated version was reviewed by the consensus panel against the original EBBS to identify and resolve potential conceptual discrepancies.

Content validity was assessed according to the COSMIN framework, considering relevance, comprehensiveness, and comprehensibility [19]. Relevance and comprehensiveness were examined through clinical/expert review and feedback from the target population. The clinical/expert review was conducted independently by two specialists, one from sport sciences and one physiotherapist, who assessed the relevance of each item to the Portuguese health and exercise context and verified whether the conceptual domains of the original EBBS were retained.

Comprehensibility was assessed through a face-to-face cognitive pre-test with students from the Polytechnic Institute of Coimbra, selected to reflect heterogeneity in sex, age, school, and degree level. Participants completed the pre-final European Portuguese version of the EBBS and then took part in a cognitive debriefing interview, guided by a questionnaire addressing difficulties in understanding the instructions, items, response options, and wording, as well as the relevance of the items and possible missing content. Any item, word, or response option reported as unclear or ambiguous was reviewed by the consensus panel before establishing the final European Portuguese version of the EBBS.

### 2.2. Validation Study

#### 2.2.1. Subjects

The target population for the validation study was higher education students enrolled at the Polytechnic Institute of Coimbra. Participants were recruited using non-probabilistic convenience sampling. Sample-size adequacy was based on the criterion for factor analysis of at least seven participants per item and a minimum sample size of 100 [23]. For the 43-item EBBS, this corresponded to a minimum required sample of 301 participants; therefore, the final sample of 324 participants met this criterion. To mitigate potential selection bias and promote diversity in academic profiles, the study was disseminated across the institution’s schools by institutional email and through in-class distribution of the questionnaire link or QR code.

The eligibility criteria were: providing informed consent, being aged 18 years or older, being actively enrolled at the Polytechnic Institute of Coimbra, and being able to read and understand Portuguese independently. Pregnant women and individuals with health conditions or medical contraindications to exercise were excluded. If consent was not provided or any eligibility criterion was not met, the online questionnaire was automatically terminated.

Data were collected online using Google Forms (www.docs.google.com/forms; accessed on 11 March 2026) between 11 March and 6 April 2026. The online form required completion of all fields before submission. Participants could discontinue the questionnaire before submission, in which case their responses were not retained. Consequently, the analytical dataset contained no item-level missing data. At baseline, corresponding to the first assessment moment (M1), participants completed the sociodemographic and academic questionnaire, the European Portuguese version of the EBBS, and previously adapted Portuguese versions of the Exercise Motivation Inventory-2 (EMI-2) [24] and the International Physical Activity Questionnaire—Short Form (IPAQ-SF) [25].

For test–retest reliability, participants who voluntarily provided an email address were invited to complete the EBBS again seven days after baseline, corresponding to the second assessment moment (M2). The email address was used only to match M1 and M2 responses and was subsequently deleted to ensure anonymisation after matching. Exercise practice was classified according to the ACSM preparticipation screening criterion for current exercise participation, defined as planned and structured physical activity of at least moderate intensity, performed for ≥30 min on ≥3 days/week during the previous 3 months [2].

#### 2.2.2. Measurements

Sociodemographic, academic, and behavioural data were collected using a questionnaire developed for this study. Variables included age, sex, nationality, place of birth, marital status, school attended, degree programme, student worker status, scholarship status, number of children, whether participants lived alone, and exercise practice.

The EBBS was originally developed by Sechrist et al. [10] to assess perceived benefits and barriers to exercise. The instrument consists of 43 items rated on a 4-point Likert scale, from 1 = strongly disagree to 4 = strongly agree. The scale includes two main dimensions: the Benefits Subscale, composed of 29 items, and the Barriers Subscale, composed of 14 items. Benefits scores range from 29 to 116, Barriers scores range from 14 to 56, and the EBBS Total score ranges from 43 to 172. For the EBBS Total score, barrier items were reverse-scored, with higher total scores indicating more favourable perceptions towards exercise.

The EMI-2 was used as a comparator instrument to assess motives for exercise participation [26]. The European Portuguese version used in this study includes 51 items organised into 14 motivational factors and scored from 0 = not at all true for me to 5 = very true for me [24]. Higher scores indicate greater endorsement of each exercise motive.

The IPAQ-SF was used to assess self-reported physical activity and sedentary behaviour during the previous seven days [27]. The questionnaire includes items on vigorous activity, moderate activity, walking, and sitting time, allowing the estimation of physical activity volume in MET-min/week and sitting time in minutes/week. Evidence for the Portuguese version of the IPAQ has been reported for both the short and long self-administered forms in adults, using accelerometry/actigraphy as the criterion measure [25].

#### 2.2.3. Statistical Analyses

Statistical analyses were performed using IBM SPSS Statistics for Windows, version 30.0 (IBM Corp., Armonk, NY, USA), and IBM SPSS Amos, version 30.0 (IBM Corp., Armonk, NY, USA), for confirmatory factor analysis. The level of statistical significance was set at *p* < 0.05. Continuous variables were summarised using means and standard deviations, and categorical variables were summarised using frequencies and percentages.

The structural validity of the Portuguese version of the EBBS was assessed through confirmatory factor analysis (CFA) by testing a second-order hierarchical model. The model was estimated using maximum likelihood estimation. The four-category EBBS items were entered as observed numerical indicators and treated as continuous for estimation purposes. This approach was used to estimate the prespecified second-order hierarchical model and evaluate the transferability of the original factorial structure. In this model, the nine first-order factors, namely Life Enhancement, Physical Performance, Psychological Outlook, Social Interaction, Preventive Health, Exercise Milieu, Time Expenditure, Physical Exertion, and Family Discouragement, were loaded onto two higher-order latent dimensions, Benefits and Barriers, in accordance with the structure proposed in the original scale development study [10]. Only this original theoretically specified model was tested; no alternative, reduced, or data-driven models were fitted. Modification indices were inspected as diagnostic information regarding potential areas of local misfit but were not used to modify the theoretically specified model. To evaluate the global fit of the model, the chi-square statistic (χ^2^), degrees of freedom (df), χ^2^/df ratio, Comparative Fit Index (CFI), Tucker–Lewis Index (TLI), Root Mean Square Error of Approximation (RMSEA) with the 90% confidence interval, and Standardized Root Mean Square Residual (SRMR) were considered. According to COSMIN criteria for structural validity based on CFA, the reference thresholds were CFI or TLI > 0.95, RMSEA < 0.06, or SRMR < 0.08 [18]. The overall pattern of fit indices was considered when interpreting structural validity, rather than relying on a single index.

Internal consistency was assessed using Cronbach’s alpha and McDonald’s omega for the Benefits Subscale, Barriers Subscale, and EBBS Total score. Coefficients ≥ 0.70 were considered adequate [18]. Corrected item–total correlations were also calculated separately for the Benefits Subscale, Barriers Subscale, and EBBS Total score. Corrected item–total correlations ≥ 0.30 were considered adequate [28]. For items presenting corrected item–total correlations below 0.30, Cronbach’s alpha and McDonald’s omega were additionally recalculated after item deletion to examine the effect on internal consistency.

Test–retest reliability was assessed in the retest subsample using a two-way random-effects, absolute-agreement, single-measure intraclass correlation coefficient, ICC(2,1), with 95% confidence intervals [29,30]. ICC values ≥ 0.70 were considered acceptable for reliability [18].

Measurement error was quantified using the standard error of measurement (SEM) and the smallest detectable change (SDC). SEM was calculated as SD × √(1 − ICC), and SDC was calculated as 1.96 × √2 × SEM [23,29]. Agreement between M1 and M2 was further examined using Bland–Altman analysis, including mean differences and 95% limits of agreement [31].

Interpretability was assessed through floor and ceiling effects, calculated as the proportion of participants obtaining the minimum or maximum possible scores for the Benefits Subscale, Barriers Subscale, and EBBS Total score. Floor or ceiling effects greater than 15% were considered potentially problematic [23,29].

Construct validity was assessed through a priori hypothesis testing. The following hypotheses were specified: H1: the Benefits Subscale and the EBBS Total score were expected to show positive correlations with the EMI-2 factors theoretically associated with exercise participation, whereas the Barriers Subscale was expected to show negative correlations or correlations of lower magnitude with these factors; H2: the Benefits Subscale and the EBBS Total score were expected to show positive correlations with the physical activity indicators assessed by the IPAQ-SF, namely vigorous, moderate, and total physical activity, whereas the Barriers Subscale was expected to show negative correlations with these indicators; H3: the Benefits Subscale and the EBBS Total score were expected to show negative correlations with sedentary time, assessed as sitting time by the IPAQ-SF, whereas the Barriers Subscale was expected to show a positive correlation with this indicator; and H4: students who met the exercise practice criterion during the previous 3 months were expected to have significantly higher scores on the Benefits Subscale and EBBS Total score, and significantly lower scores on the Barriers Subscale, than students who did not meet this criterion. Correlation-based hypotheses were examined using Spearman’s rank correlation coefficients between the EBBS scores and the EMI-2 factors and IPAQ-SF variables. To assess the potential influence of multiplicity, a sensitivity analysis was conducted using the Benjamini–Hochberg procedure to control the false discovery rate at 5% [32]. The correction was applied separately to the EMI-2 and IPAQ-SF families of correlations, reflecting their distinct comparator constructs. The magnitude of Spearman’s correlation coefficients was interpreted using absolute values as follows: very high correlation, ≥0.90; high correlation, 0.70–0.89; moderate correlation, 0.40–0.69; low correlation, 0.20–0.39; and very low correlation, ≤0.19 [33]. Known-groups validity was tested by comparing EBBS scores between students who met the exercise practice criterion and those who did not. Between-group comparisons were performed using the Mann–Whitney U test. Rank-biserial correlations were calculated as effect-size measures for the Mann–Whitney comparisons using *r*_rb_ = 1 − 2*U*/(*n*_1_*n*_2_), where *U* is the Mann–Whitney statistic and *n*_1_ and *n*_2_ are the respective group sizes [34].

## 3. Results

### 3.1. Translation, Cross-Cultural Adaptation and Content Validity

The two independent forward translations showed overall convergence, with differences mainly related to lexical choices. During the synthesis and consensus process, the panel selected the wording considered most appropriate for European Portuguese while preserving the meaning of the original items.

Item 28 of the original scale, “I think people in exercise clothes look funny”, required specific discussion because the initial translation proposals conveyed different nuances. Given that the item belongs to the Barriers Subscale in the original EBBS, the final Portuguese wording was defined as “Acho que as pessoas com roupa desportiva ficam estranhas”. The back-translation was reviewed against the original version, and no conceptual discrepancies requiring structural modification of the scale were identified.

The clinical/expert review and feedback from the target population during cognitive pre-testing indicated the relevance of the items. Regarding comprehensiveness, no missing conceptual domains were identified during the clinical/expert review or reported during cognitive pre-testing. The original 43-item structure, including the Benefits and Barriers dimensions, was therefore retained.

Comprehensibility was assessed through a face-to-face cognitive pre-test with 16 students. Participants had a mean age of 20.63 ± 2.33 years and included students from five of the six schools, both sexes, and different degree levels. During the pre-test, no additional difficulties were identified regarding the instructions or item wording, except for the term “tónus” in item 17, which was not understood by 37.5% of participants, none of whom were from health- or sport-related fields. To improve clarity, the term “firmeza” was added in parentheses, resulting in the wording “O meu tónus (firmeza) muscular melhora com o exercício”.

Two participants also reported difficulty interpreting the response format when abbreviated response labels were used. Accordingly, the response layout was changed to checkbox-style options. The mean completion time for the European Portuguese version of the EBBS was 2 min 58 s ± 32 s. Following these adjustments, the final European Portuguese version of the EBBS was established for the validation study.

### 3.2. Validation Study

Of the 371 individuals initially recruited, 324 met the eligibility criteria and were included in the validation sample. Of these, 72 completed the EBBS a second time seven days after the initial administration. Sociodemographic, academic, and behavioural characteristics of the total sample and reproducibility subsample are presented in Table 1. In the total sample, the mean age was 21.78 ± 5.74 years, 71.0% were female, and 68.5% met the ACSM exercise practice criterion.

Descriptive statistics for the EBBS and comparator instruments are presented in Table 2. Mean EBBS scores in the total sample and reproducibility subsample were, respectively, 97.44 ± 11.53 and 98.18 ± 10.09 for Benefits, 28.68 ± 6.32 and 27.82 ± 4.75 for Barriers, and 138.76 ± 14.82 and 140.36 ± 12.30 for the Total score.

Structural validity was examined using a second-order hierarchical CFA model. Only the original theoretically specified model was tested, and no alternative, reduced, or data-driven models were fitted. Complete CFA results are provided in the Supplementary Materials, including standardized factor loadings, residual variances, the correlation and covariance between the second-order latent factors, and modification indices (Tables S1–S4). As shown in Table 3, the model yielded χ^2^(851) = 2137.819, *p* < 0.001, χ^2^/df = 2.512, CFI = 0.808, TLI = 0.797, RMSEA = 0.068, 90% CI = 0.065–0.072, and SRMR = 0.0774.

Internal consistency, test–retest reliability, and measurement error results are summarised in Table 4. Cronbach’s alpha and McDonald’s omega were, respectively, 0.944 and 0.944 for the Benefits Subscale, 0.831 and 0.824 for the Barriers Subscale, and 0.926 and 0.920 for the EBBS Total score. ICC values were 0.817 for Benefits, 0.784 for Barriers, and 0.847 for the EBBS Total score. SEM values ranged from 2.21 to 4.81, and SDC values ranged from 6.12 to 13.33.

Corrected item–total correlations are presented in Supplementary Table S5. Values ranged from 0.175 to 0.748 for the Benefits Subscale, from 0.358 to 0.560 for the Barriers Subscale, and from 0.065 to 0.718 for the EBBS Total score. For the EBBS Total score, Items 4, 9, 24, 28, and 39 presented corrected item–total correlations below 0.30 (0.244, 0.259, 0.246, 0.277, and 0.065, respectively). Item 39 also presented a corrected item–total correlation below 0.30 for the Benefits Subscale (0.175). Recalculation after individual item deletion showed no meaningful improvement in internal consistency for Items 4, 9, 24, or 28. Deleting Item 39 produced the largest, although still small, increases: Cronbach’s alpha increased from 0.944 to 0.948 for the Benefits Subscale and from 0.926 to 0.929 for the EBBS Total score, while McDonald’s omega increased from 0.944 to 0.949 and from 0.920 to 0.924, respectively.

**Figure 1:**
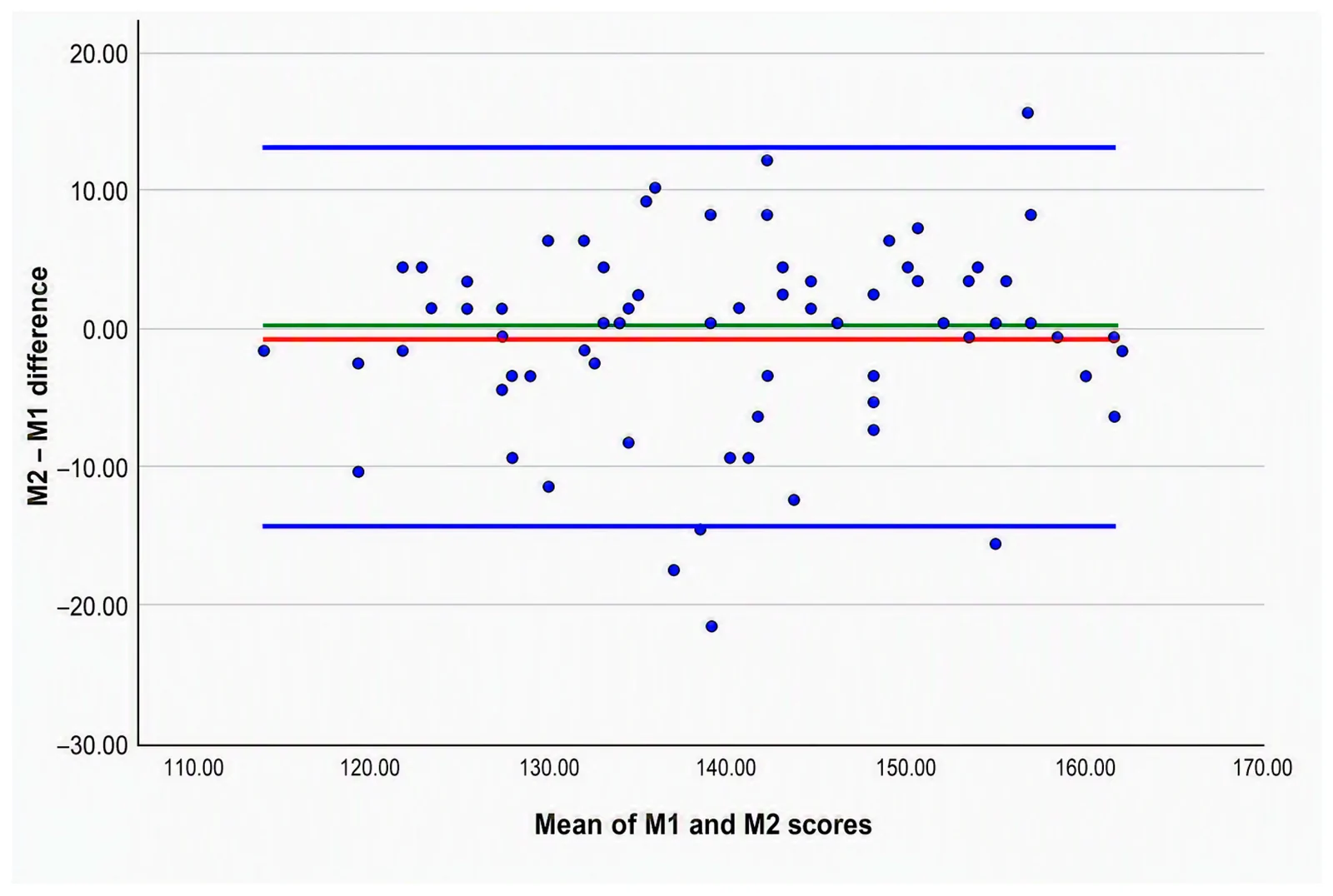
Bland–Altman plot for the EBBS Total score. The green line represents zero difference between M2 and M1, the red line represents the mean difference (bias), and the blue lines represent the 95% limits of agreement.

Bland–Altman results are reported in Table 4. The Bland–Altman plot for the EBBS Total score is presented in Figure 1, whereas the corresponding plots for the Benefits and Barriers Subscales are provided in the Supplementary Materials as Figures S1 and S2, respectively. Mean differences between M2 and M1 were −1.653 for the Benefits Subscale, −0.556 for the Barriers Subscale, and −1.097 for the EBBS Total score. The corresponding limits of agreement were −13.307 to 10.002, −6.839 to 5.728, and −14.761 to 12.566, respectively.

Floor and ceiling effects are shown in Table 5. No floor or ceiling effects were observed for the EBBS Total score. For the Benefits Subscale, a ceiling effect was observed in eight participants (2.5%), with no floor effect. For the Barriers Subscale, a floor effect was observed in one participant (0.3%), with no ceiling effect.

Correlation-based construct validity hypothesis testing is presented in Table 6. For the EBBS Total score, correlations with EMI-2 were moderate for Enjoyment (ρ = 0.606), Revitalisation (ρ = 0.545), Challenge (ρ = 0.482), Strength and Endurance (ρ = 0.498), Nimbleness (ρ = 0.457), Positive Health (ρ = 0.442), and Stress Management (ρ = 0.418). Correlations were very low for Social Recognition (ρ = 0.080), Health Pressures (ρ = 0.005), Weight Management (ρ = 0.141), and Appearance (ρ = 0.150). Correlations with IPAQ-SF variables ranged from ρ = −0.174 to ρ = 0.359 for the EBBS Total score; walking showed correlations ranging from ρ = −0.012 to ρ = 0.051 across the three EBBS scores. In the sensitivity analysis, all 36 EMI-2 correlations and all 12 IPAQ-SF correlations that were statistically significant using the unadjusted *p*-values remained significant after Benjamini–Hochberg correction at a false discovery rate of 5%.

Known-group comparisons are presented in Table 7. Students who met the ACSM exercise practice criterion had higher mean ranks for the Benefits Subscale and EBBS Total score, and lower mean ranks for the Barriers Subscale than those who did not. All comparisons were statistically significant, with *p* < 0.001. The absolute rank-biserial correlations were 0.466 for the Benefits Subscale, 0.393 for the Barriers Subscale, and 0.526 for the EBBS Total score.

## 4. Discussion

This study aimed to translate and cross-culturally adapt the EBBS into European Portuguese and to evaluate its psychometric properties in higher education students. The translation and adaptation process preserved the conceptual content of the original instrument, while minor linguistic and layout adjustments improved comprehensibility for students from different academic backgrounds. Psychometric testing provided favourable evidence for content validity, internal consistency, test–retest reliability, interpretability, and several aspects of construct validity, and provided estimates of measurement error, whereas the original 43-item second-order factorial structure received only partial and limited support. Taken together, these findings support the potential use of the European Portuguese EBBS in higher education students, although the factorial structure and the extent to which the EBBS Total score can be meaningfully interpreted require further evaluation.

The cross-cultural adaptation process highlighted the importance of moving beyond literal translation when adapting self-report health measurement instruments. Most items showed semantic convergence across translations, but specific lexical choices required careful consideration to preserve the intended conceptual meaning. The discussion around item 28, originally phrased as “I think people in exercise clothes look funny”, illustrates this issue particularly well. In the original EBBS, this item belongs to the Barriers Subscale; therefore, the European Portuguese wording needed to retain a connotation of perceived discomfort or negative appraisal rather than humour or amusement. Similarly, the clarification of the term “tónus” by adding “firmeza” in item 17 reflected the need to maintain technical accuracy while ensuring that the item remained understandable to students outside health- or sport-related fields. These findings are consistent with established recommendations that cross-cultural adaptation should preserve conceptual, semantic, and operational equivalence while ensuring comprehensibility in the target population [16,17]. They also reinforce the centrality of content validity and cognitive pre-testing in the adaptation of self-report health measures [19].

The structural validity analysis provided only partial and limited support for the original theoretical organisation of the EBBS. The tested second-order hierarchical model reflected the conceptual structure proposed by Sechrist et al. [10], in which first-order benefit and barrier domains load onto broader latent dimensions of perceived benefits and perceived barriers. Although the SRMR met the prespecified COSMIN criterion of < 0.08, the CFI and TLI were substantially below the reference threshold of 0.95, and the RMSEA exceeded the prespecified criterion of < 0.06 [18]. Considered together, these findings indicate that the original 43-item second-order factorial structure was not adequately confirmed in this sample. Such findings are not unexpected. Previous studies have reported variations in the EBBS factorial structure across cultural and population contexts, including reduced or alternative factor configurations [11,12]. Koehn and Amirabdollahian [12], for example, proposed a more concise 26-item structure after confirmatory analyses in a UK sample, suggesting that some original items or domains may be less psychometrically informative in contemporary or specific cultural contexts.

An exploratory factor analysis was not conducted because splitting the sample would have resulted in approximately 162 participants for model development and 162 for model confirmation, which was considered insufficient for independently deriving and confirming a potentially complex structure comprising 43 items and nine first-order factors. Furthermore, the primary purpose of this study was to evaluate the transferability of the original theoretically specified EBBS structure rather than to derive a new or reduced European Portuguese version. Conducting iterative exploratory and confirmatory analyses using the same full sample would not have provided independent confirmation of an alternative structure and could have produced sample-specific or potentially misleading solutions [35]. Similarly, although modification indices were inspected and are reported in Supplementary Table S4, no suggested parameters were added because post hoc modifications based on the present sample would have introduced data-driven changes without independent confirmation. The decision to retain the full 43-item version was nevertheless justified at this stage of adaptation. The original EBBS was developed to capture a broad theoretical construct, encompassing multiple perceived benefits and barriers linked to exercise behaviour [10]. Moreover, previous cross-cultural studies have evaluated translated versions of the EBBS while retaining the broad distinction between perceived benefits and perceived barriers, supporting its usefulness across different populations and linguistic contexts [13,14]. Preserving the full version also facilitates comparability with earlier validation studies and allows future research to determine whether item reduction is warranted in European Portuguese samples. Given that this is the first European Portuguese adaptation of the EBBS, retaining the original item set reduces the risk of prematurely excluding content that may be relevant in other Portuguese-speaking populations, clinical groups, or age ranges. At the same time, evidence from studies proposing more concise EBBS structures indicates that alternative models and shorter forms should be examined in future research rather than assumed a priori [12]. Future studies using larger and more heterogeneous samples should therefore further examine the factorial structure, including alternative models, measurement invariance across sex and academic fields, and potential shorter forms.

Cronbach’s alpha and McDonald’s omega were above the predefined adequacy threshold for the EBBS Total score and both subscales. The similar values obtained with both coefficients provide convergent information regarding the internal consistency of the scores. However, these coefficients should be interpreted cautiously because the original factorial structure received only partial and limited support, and internal consistency does not constitute evidence of structural validity. Accordingly, neither Cronbach’s alpha nor McDonald’s omega alone justifies interpreting the EBBS Total score as a valid unidimensional measure of exercise-related perceptions. Moreover, very high coefficients may reflect item redundancy, particularly in broad multidimensional constructs [23]. These findings are consistent with prior EBBS studies reporting satisfactory reliability across different populations and cultural settings [11,12,13,14].

The corrected item–total correlations provided additional information on the functioning of specific items. Although Items 4, 9, 24, and 28 also presented corrected item–total correlations below 0.30 for the EBBS Total score, their individual deletion did not meaningfully change Cronbach’s alpha or McDonald’s omega. Item 39, referring to being better accepted by others because of exercise, showed notably low corrected item–total correlations with both the Benefits Subscale (0.175) and the EBBS Total score (0.065). Although no comprehension difficulties were identified during cognitive pre-testing, the notion that exercise leads to greater acceptance by others may have limited relevance for Portuguese higher education students. The item may also be interpreted in different ways, including social approval, interpersonal belonging, or acceptance based on physical appearance. This conceptual ambiguity may distinguish Item 39 from other social-benefit items that refer more directly to contact with friends or meeting new people. This interpretation is consistent with the very low and non-significant correlation between the EMI-2 Social Recognition factor and the EBBS Total score. Deleting Item 39 resulted in only small increases in internal consistency: Cronbach’s alpha increased from 0.944 to 0.948 for the Benefits Subscale and from 0.926 to 0.929 for the EBBS Total score, while McDonald’s omega increased from 0.944 to 0.949 and from 0.920 to 0.924, respectively. These changes did not meaningfully alter the already high internal consistency of either score. Item 39 was therefore retained to preserve the content coverage and comparability of the original 43-item EBBS. Nevertheless, its interpretation, factor loading, differential item functioning, and performance in larger and more diverse European Portuguese samples should be examined before deciding whether rewording or removal is justified. The limited relevance of social acceptance as a perceived exercise benefit in this sample may be interpreted within the Health Promotion Model according to which individuals are more likely to commit to behaviours from which they anticipate personally valued benefits [7,8].

Test–retest reliability findings support the temporal stability of the European Portuguese EBBS over a 7-day interval. ICC values indicate acceptable reliability for the total score and both subscales according to the predefined criterion, suggesting that the instrument can produce stable scores when the construct is expected to remain unchanged. The 7-day retest interval reduced the likelihood of genuine changes in exercise perceptions; however, it may have increased the possibility that participants remembered their previous responses, potentially inflating the reliability estimates [29]. Although all participants completed the retest seven days after the initial assessment, future studies should evaluate test–retest reliability over a longer interval, such as 2–4 weeks. The measurement-error estimates provide reference values for interpreting individual change in future longitudinal and intervention studies. The SEM values of 4.32 points for the Benefits Subscale, 2.21 points for the Barriers Subscale, and 4.81 points for the EBBS Total score reflect the measurement uncertainty associated with an observed score. At the individual level, changes must exceed the corresponding SDC values of 11.96, 6.12, and 13.33 points, respectively, to be distinguished from measurement error with 95% confidence. Changes equal to or below these thresholds cannot be confidently interpreted as genuine individual change. However, the SDC indicates detectable change beyond measurement error and should not be interpreted as evidence that a change is clinically or practically meaningful. Future responsiveness studies are required to establish thresholds for meaningful change. The Bland–Altman analyses showed small mean differences between test and retest assessments. For the Benefits Subscale, the mean difference between M2 and M1 was −1.653 points (95% CI: −3.050 to −0.256), indicating a small systematic mean shift towards lower scores at retest. In contrast, the 95% confidence intervals for the Barriers Subscale and the EBBS Total score included zero, providing no clear evidence of systematic mean shifts for these scores.

These findings are particularly relevant because measurement error is often underreported in validation studies, despite being essential for interpreting change over time [23,29].

Interpretability was also supported by the absence of problematic floor or ceiling effects. The EBBS Total score showed no floor or ceiling effects and the subscale effects were negligible. This suggests that the instrument can discriminate between students with different levels of perceived benefits and barriers rather than clustering responses at the extremes. Adequate interpretability is important for both research and applied contexts because instruments with substantial floor or ceiling effects may be insensitive to differences between individuals or to changes following interventions [23]. In this sample, the relatively high Benefits scores and lower Barriers scores indicate generally favourable perceptions toward exercise, but without compromising the scale’s ability to capture variability.

Construct validity findings were heterogeneous, with stronger evidence for the physical activity, sedentary behaviour, and known-group hypotheses than for the EMI-2 motivational dimensions. The expected associations were most clearly supported for Enjoyment, Revitalisation, Challenge, Stress Management, Strength and Endurance, Nimbleness, and Positive Health. Conversely, Social Recognition and Health Pressures showed no meaningful associations with the EBBS Total score, whereas Weight Management and Appearance showed only very low associations. Accordingly, the EMI-2 findings should be interpreted as providing partial and dimension-dependent, rather than uniformly supportive, evidence of construct validity.

The EMI-2 was selected because exercise motives are theoretically related to perceived exercise benefits and barriers, although the two instruments assess distinct constructs and the EMI-2 factors are not equally close conceptual comparators for every EBBS dimension. The stronger associations with Enjoyment and Revitalisation suggest that favourable exercise perceptions in this sample were more closely related to intrinsic and experiential motives than to Social Recognition, Health Pressures, Weight Management, or Appearance. This interpretation remains consistent with the Health Promotion Model, in which perceived benefits and barriers are behaviour-specific cognitions relevant to health-promoting behaviour [8], and with evidence linking positive outcome expectancies to physical activity [9]. Associations between specific EBBS factors and conceptually corresponding EMI-2 factors were not examined because these analyses were not prespecified and the original factorial structure received only limited support.

The associations with IPAQ-SF indicators provided additional support for construct validity, although the magnitude of correlations was generally low to moderate. Higher Benefits and EBBS Total scores were associated with greater vigorous, moderate, and total physical activity, whereas higher Barriers scores were associated with lower physical activity and greater sitting time. These findings are theoretically coherent: individuals who perceive exercise as beneficial and less obstructed are more likely to report higher activity levels, whereas those perceiving more barriers are more likely to report lower activity and more sedentary behaviour. The magnitude of these correlations should not be interpreted as a weakness of the EBBS. Exercise behaviour is multidetermined, and self-reported physical activity is influenced by measurement error, recall bias, social desirability, and contextual constraints [27]. In addition, the EBBS assesses perceptions specifically related to exercise, whereas the IPAQ-SF captures broader physical activity domains over the previous seven days. This conceptual mismatch may partially explain the modest associations, particularly for walking, which did not correlate significantly with EBBS scores and may be perceived more as transportation or daily activity than as structured exercise.

Known-group validity was supported by consistent between-group differences and the corresponding rank-biserial effect sizes. Students who met the exercise practice criterion had more favourable EBBS profiles than those who did not, with higher Benefits and Total scores and lower Barriers scores. The absolute rank-biserial correlations were 0.466 for the Benefits Subscale, 0.393 for the Barriers Subscale, and 0.526 for the EBBS Total score, indicating that the observed differences were not limited to statistical significance. This finding is consistent with the theoretical expectation that perceived benefits and barriers differentiate individuals according to exercise behaviour. It also supports the practical relevance of the EBBS in higher education settings, where identifying perceptual profiles may help inform targeted interventions. For example, students with high perceived barriers may benefit from strategies addressing time management, access, fatigue, or confidence, whereas students with lower motivational endorsement may benefit from interventions that enhance enjoyment, social support, and perceived immediate gains. Such approaches are consistent with contemporary evidence that university students’ physical activity is shaped by environmental resources, goals, social influences, motivation, and perceived consequences [4,5].

The findings have several scientific and practical implications. Scientifically, this study provides the first evidence supporting the use of a European Portuguese version of the EBBS in higher education students, addressing an important gap in exercise-related measurement in Portugal. The instrument may support future research examining perceptual determinants of exercise, intervention responsiveness, and cross-cultural comparisons. Practically, the EBBS may be useful for educational, institutional, and health-promotion contexts by helping identify perceived benefits and barriers that are likely to influence exercise engagement. Although this study was not conducted in a clinical population, the scale may also have future relevance for physiotherapy and rehabilitation contexts, where understanding perceived barriers and benefits can inform exercise prescription, adherence strategies, and patient-centred counselling. However, such applications require further validation in clinical samples before broader implementation.

This study has several strengths. The translation and adaptation process followed internationally recognised procedures and included expert review and cognitive pre-testing with the target population. The psychometric evaluation was multidimensional, including structural validity, internal consistency, corrected item–total correlations, test–retest reliability, measurement error, Bland–Altman agreement, interpretability, and construct validity through predefined hypotheses. The inclusion of both motivational and behavioural comparator instruments strengthened construct validity assessment by linking perceptions of exercise to theoretically related constructs and self-reported activity patterns. The use of a retest subsample also allowed the estimation of temporal stability and measurement error, which are particularly important for future longitudinal or intervention studies.

Several limitations should nevertheless be acknowledged. First, although the overall sample size met the prespecified adequacy criterion, the tested model was complex, comprising 43 observed variables, nine first-order factors, and two second-order factors. The sample was recruited by convenience from a single higher education institution and was predominantly female (71.0%), with a substantial proportion of students from the Health School (58.6%). This limited heterogeneity may have influenced the estimated relationships among the items and factors, potentially reducing the stability and generalisability of the factorial solution to more balanced or academically diverse populations. Measurement invariance across sex and academic field was not evaluated, and subgroup-specific fit indices were not estimated, because the sex subgroups were markedly unequal (230 female and 94 male participants) and the original factorial structure was not adequately confirmed in the full sample. A sensitivity analysis restricted to students outside the Health School was also not conducted because it would have included only 134 participants, which was considered insufficient for the complexity of the tested model. Second, all behavioural and perceptual data were collected through self-report instruments, increasing the possibility of recall bias and social desirability bias. The mandatory-response format may have discouraged questionnaire completion or influenced responses to questions perceived as personal, introducing potential completion or response bias. Future studies should permit participants to omit questions they prefer not to answer and should prespecify an appropriate strategy for handling missing data. The IPAQ-SF is widely used and has established international evidence, but self-reported physical activity may overestimate actual activity levels when compared with objective measures [27]. Third, the cross-sectional design limits causal interpretation. Although EBBS scores were associated with motivation, activity indicators, and exercise practice status, these associations do not establish whether perceptions influence exercise behaviour, whether exercise behaviour modifies perceptions, or whether both are shaped by other individual and contextual factors. Fourth, responsiveness was not evaluated, and therefore the ability of the European Portuguese EBBS to detect change after intervention remains unknown. In addition, the 7-day test–retest interval may have increased the possibility of response recall and consequently inflated the reliability estimates, despite all participants completing the retest at the intended interval. Future studies should examine test–retest reliability over longer intervals, such as 2–4 weeks, to reduce the potential influence of recall and strengthen the evidence regarding temporal stability. Because the four-category EBBS items were treated as continuous for estimation purposes, the CFA findings may have been sensitive to the estimation approach. Future studies should examine the factorial structure using estimators specifically designed for ordinal indicators. Finally, the overall pattern of CFA fit indices provided only partial and limited support for the original 43-item second-order factorial structure, which was not adequately confirmed in this sample. The absence of EFA, exploratory structural equation modelling (ESEM), and alternative-model testing should also be acknowledged as a limitation. A bifactor model was not evaluated; therefore, the extent to which the EBBS Total score reflects a sufficiently strong general factor remains unknown.

Future research should address these limitations by testing the European Portuguese EBBS in larger, multicentre, and more sociodemographically diverse samples with a more balanced representation across sex and academic fields. Such studies should evaluate measurement invariance and subgroup-specific model fit across sex, academic field, exercise status, and other relevant subgroups. Alternative and reduced factorial structures, including a theoretically justified bifactor model, should be investigated and independently confirmed in larger and more heterogeneous European Portuguese samples. Bifactor-specific indices, including omega hierarchical and explained common variance, should be examined before supporting interpretation of the EBBS Total score as a unidimensional measure. After the EBBS factorial structure has been independently confirmed, future studies should test explicitly prespecified associations between specific EBBS factors and conceptually corresponding EMI-2 factors. Longitudinal studies are needed to assess responsiveness and to determine whether changes in perceived benefits and barriers predict changes in exercise behaviour over time. Future work should also include objective physical activity measures, such as accelerometry, to complement self-report data and clarify the relationship between perceived exercise-related cognitions and actual movement behaviour. Finally, validation in clinical and rehabilitation populations would be valuable before using the EBBS as a decision-support tool in physiotherapy or other health-care contexts.

Overall, these findings provide a coherent interpretation of the measurement properties of the European Portuguese EBBS in higher education students. They support its potential use in research and health-promotion contexts, while also indicating that further work is needed to refine the evidence on its factorial structure and extend validation to other populations.

## 5. Conclusions

This study translated, cross-culturally adapted, and psychometrically evaluated the EBBS into European Portuguese for use with higher education students in Portugal. The European Portuguese EBBS is a culturally appropriate instrument with favourable evidence for several measurement properties; however, its original 43-item second-order factorial structure was not adequately confirmed in this sample.

The study provides the first European Portuguese version of the EBBS and addresses an important measurement gap in exercise-related research in Portugal. Its use may help researchers, educators, and health-promotion professionals identify exercise-related perceptions that are relevant to participation in structured exercise among higher education students. Future studies should further examine the factorial structure, responsiveness, and applicability of the instrument in more diverse and clinical populations.

## Figures and Tables

**Table 1:** Sociodemographic characteristics of the total sample and reproducibility subsample.

Characteristics	Total Sample (*N* = 324)	Reproducibility Subsample (*n* = 72) *
**Sex**		
Female	230 (71.0%)	59 (81.9%)
Male	94 (29.0%)	13 (18.1%)
Age (years)	21.78 ± 5.74	21.78 ± 4.84
**Nationality**		
Portuguese	305 (94.1%)	71 (98.6%)
Other	19 (5.9%)	1 (1.4%)
**Place of birth**		
North	41 (12.7%)	10 (13.9%)
Center	187 (57.8%)	48 (66.7%)
West and Tagus Valley	37 (11.4%)	6 (8.3%)
Greater Lisbon	5 (1.5%)	2 (2.8%)
Setúbal Peninsula	3 (0.9%)	—
Alentejo	3 (0.9%)	1 (1.4%)
Algarve	5 (1.5%)	3 (4.2%)
Autonomous Region of the Azores	12 (3.7%)	—
Autonomous Region of Madeira	7 (2.2%)	1 (1.4%)
Foreign-born	24 (7.4%)	1 (1.4%)
**Marital status**		
Single	308 (95.1%)	70 (97.2%)
Married	8 (2.5%)	—
Civil partnership	6 (1.9%)	1 (1.4%)
Divorced	2 (0.6%)	1 (1.4%)
**School attended**		
Coimbra Agriculture School	14 (4.3%)	2 (2.8%)
Coimbra Education School	34 (10.5%)	9 (12.5%)
Oliveira do Hospital School of Technology and Management	4 (1.2%)	1 (1.4%)
Coimbra Health School	190 (58.6%)	48 (66.7%)
Coimbra Business School	55 (17.0%)	5 (6.9%)
Coimbra Engineering School	27 (8.3%)	7 (9.7%)
**Degree programme**		
Short-cycle higher technical programme	3 (0.9%)	1 (1.4%)
Bachelor’s degree	279 (86.1%)	60 (83.3%)
Master’s degree	34 (10.5%)	10 (13.9%)
Postgraduate programme	8 (2.5%)	1 (1.4%)
**Student worker status**		
Yes	48 (14.8%)	11 (15.3%)
No	276 (85.2%)	61 (84.7%)
**Scholarship**		
Yes	110 (34.0%)	30 (41.7%)
No	214 (66.0%)	42 (58.3%)
**Number of children**		
0	315 (97.2%)	71 (98.6%)
1	3 (0.9%)	1 (1.4%)
2	6 (1.9%)	—
**Living alone**		
Yes	46 (14.2%)	6 (8.3%)
No	278 (85.8%)	66 (91.7%)
**Exercise practice according to ACSM criterion**		
Yes	222 (68.5%)	48 (66.7%)
No	102 (31.5%)	24 (33.3%)

Note. Continuous variables are expressed as mean ± standard deviation; categorical variables are expressed as frequency (percentage). * Participants who completed the questionnaire for a second time, seven days after the initial administration. Exercise practice was classified according to the ACSM criterion, defined as planned and structured physical activity of at least moderate intensity, performed for ≥30 min on ≥3 days per week, for at least the last 3 months.

**Table 2:** Descriptive statistics for the EBBS and comparator instruments.

Instrument/Variable	Total Sample (*N* = 324)	Reproducibility Subsample (*n* = 72) *
**EBBS**		
Benefits	97.44 ± 11.53	98.18 ± 10.09
Barriers	28.68 ± 6.32	27.82 ± 4.75
Total	138.76 ± 14.82	140.36 ± 12.30
**EMI-2**		
Stress management	3.44 ± 1.20	3.44 ± 1.14
Revitalisation	3.83 ± 1.07	3.94 ± 0.90
Enjoyment	3.87 ± 1.20	3.97 ± 1.05
Challenge	3.45 ± 1.23	3.48 ± 1.03
Social recognition	1.83 ± 1.45	1.56 ± 1.32
Affiliation	2.87 ± 1.40	2.85 ± 1.40
Competition	2.82 ± 1.61	2.77 ± 1.64
Health pressures	2.08 ± 1.46	2.12 ± 1.58
Ill-health avoidance	3.70 ± 1.15	3.74 ± 1.07
Positive health	4.27 ± 0.99	4.38 ± 0.82
Weight management	3.34 ± 1.38	3.38 ± 1.30
Appearance	3.25 ± 1.22	3.26 ± 1.13
Strength and endurance	3.97 ± 1.08	4.10 ± 0.98
Nimbleness	3.63 ± 1.26	3.64 ± 1.24
**IPAQ-SF**		
Vigorous activity (MET-min/week)	1716.30 ± 1768.13	1570.56 ± 1234.42
Moderate activity (MET-min/week)	815.00 ± 1022.09	553.89 ± 765.00
Walking (MET-min/week)	1216.81 ± 1227.31	1163.02 ± 1210.12
Total physical activity (MET-min/week)	3748.11 ± 3082.34	3287.47 ± 2180.48
Sitting time (min/week)	2586.61 ± 1102.47	2741.67 ± 965.81

Note. Values are expressed as mean ± standard deviation. * Participants who completed the questionnaire for a second time, seven days after the initial administration. EBBS = Exercise Benefits/Barriers Scale; EMI-2 = Exercise Motivation Inventory-2; IPAQ-SF = International Physical Activity Questionnaire—Short Form; MET = metabolic equivalent of task. EBBS Benefits Subscale scores range from 29 to 116; EBBS Barriers Subscale scores range from 14 to 56; EBBS Total scores range from 43 to 172. Higher EBBS Total scores indicate more favourable perceptions towards exercise, as Barriers Subscale values are reverse-scored for the total score. EMI-2 factors are scored from 0 to 5, with higher scores indicating greater endorsement of each motive. IPAQ-SF physical activity scores are expressed as MET-min/week; higher values indicate greater physical activity volume/intensity. Sitting time is expressed in minutes/week and should be interpreted inversely.

**Table 3:** Structural validity fit indices for the second-order hierarchical CFA model of the EBBS.

Fit Index	Value
χ^2^ (851)	2137.819
*p*-value	<0.001
χ^2^/df	2.512
CFI	0.808
TLI	0.797
RMSEA	0.068
90% CI for RMSEA	0.065–0.072
SRMR	0.0774

Note. EBBS = Exercise Benefits/Barriers Scale; CFA = confirmatory factor analysis; χ^2^ = chi-square; df = degrees of freedom; CFI = Comparative Fit Index; TLI = Tucker–Lewis Index; RMSEA = Root Mean Square Error of Approximation; CI = confidence interval; SRMR = Standardized Root Mean Square Residual. The tested model was a second-order hierarchical CFA model.

**Table 4:** Internal consistency, test–retest reliability, and measurement error of the EBBS.

EBBS (No. of Items)	Cronbach’s α	McDonald’s ω	ICC (95% CI) *	SEM (95% CI) *	SDC (95% CI) *	Bland–Altman Mean Difference M2 − M1 Bias (95% CI) *	Bland–Altman LoA *
EBBS Benefits (29)	0.944	0.944	0.817 (0.717–0.883)	4.32 (3.45–5.37)	11.96 (9.57–14.88)	−1.653 (−3.050; −0.256)	−13.307 to 10.002
EBBS Barriers (14)	0.831	0.824	0.784 (0.677–0.859)	2.21 (1.79–2.70)	6.12 (4.95–7.49)	−0.556 (−1.309; 0.198)	−6.839 to 5.728
EBBS Total (43)	0.926	0.920	0.847 (0.767–0.902)	4.81 (3.85–5.94)	13.33 (10.67–16.45)	−1.097 (−2.735; 0.541)	−14.761 to 12.566

Note. EBBS = Exercise Benefits/Barriers Scale; CI = confidence interval; ICC = intraclass correlation coefficient; SEM = standard error of measurement; SDC = smallest detectable change; M1 = first assessment; M2 = second assessment; LoA = limits of agreement. Internal consistency was assessed in the total sample (*N* = 324). * Test–retest reliability and measurement error were assessed in the retest subsample participants (*n* = 72), who completed the EBBS again seven days after the first assessment.

**Table 5:** Interpretability of the EBBS through floor and ceiling effects.

EBBS Score	Floor Effect, *n* (%)	Ceiling Effect, *n* (%)
Benefits Subscale	0 (0.0%)	8 (2.5%)
Barriers Subscale	1 (0.3%)	0 (0.0%)
EBBS Total	0 (0.0%)	0 (0.0%)

Note. EBBS = Exercise Benefits/Barriers Scale. Benefits Subscale scores range from 29 to 116; Barriers Subscale scores range from 14 to 56; EBBS Total scores range from 43 to 172. Floor and ceiling effects represent the number and percentage of participants obtaining the minimum and maximum possible scores, respectively. All analyses were based on *N* = 324, with no missing data.

**Table 6:** Construct validity hypothesis testing through correlations between the EBBS and comparator instruments.

Comparator Instrument/Variable	Benefits Subscale ρ	Benefits Subscale *p*-Value	Barriers Subscale ρ	Barriers Subscale *p*-Value	EBBS Total Ρ	EBBS Total *p*-Value
**EMI-2 subscales**						
Stress management	**0.457** **	<0.001	**−0.163** **	0.003	**0.418** **	<0.001
Revitalisation	**0.553** **	<0.001	**−0.275** **	<0.001	**0.545** **	<0.001
Enjoyment	**0.598** **	<0.001	**−0.339** **	<0.001	**0.606** **	<0.001
Challenge	**0.510** **	<0.001	**−0.220** **	<0.001	**0.482** **	<0.001
Social recognition	**0.156** **	0.005	0.077	0.169	0.080	0.150
Affiliation	**0.358** **	<0.001	**−0.176** **	0.001	**0.344** **	<0.001
Competition	**0.409** **	<0.001	**−0.120** *	0.032	**0.366** **	<0.001
Health pressures	0.077	0.165	0.148 **	0.008	0.005	0.922
Ill-health avoidance	**0.385** **	<0.001	**−0.147** **	0.008	**0.360** **	<0.001
Positive health	**0.449** **	<0.001	**−0.225** **	<0.001	**0.442** **	<0.001
Weight management	**0.171** **	0.002	−0.031	0.581	**0.141** *	0.011
Appearance	**0.205** **	<0.001	0.009	0.871	**0.150** **	0.007
Strength and endurance	**0.513** **	<0.001	**−0.231** **	<0.001	**0.498** **	<0.001
Nimbleness	**0.485** **	<0.001	**−0.193** **	<0.001	**0.457** **	<0.001
**IPAQ-SF**						
Vigorous activity (MET-min/week)	**0.320** **	<0.001	**−0.265** **	<0.001	**0.359** **	<0.001
Moderate activity (MET-min/week)	**0.247** **	<0.001	**−0.125** *	0.025	**0.240** **	<0.001
Walking (MET-min/week)	−0.006	0.918	0.051	0.358	−0.012	0.830
Total physical activity (MET-min/week)	**0.225** **	<0.001	**−0.153** **	0.006	**0.246** **	<0.001
Sitting time (min/week)	**−0.158** **	0.004	**0.156** **	0.005	**−0.174** **	0.002

Note. EBBS = Exercise Benefits/Barriers Scale; EMI-2 = Exercise Motivation Inventory-2; IPAQ-SF = International Physical Activity Questionnaire—Short Form; MET = metabolic equivalent of task; ρ = Spearman’s rank correlation coefficient; *p*-value = two-tailed probability value. All correlations were based on *N* = 324. Bold values indicate statistically significant correlations in the direction specified by the corresponding a priori hypothesis. ** *p* < 0.01; * *p* < 0.05.

**Table 7:** Construct validity hypotheses testing through known-group comparison.

EBBS Score	Exercise Practice According to ACSM Criterion	*N*	Mean Rank	Mann–Whitney U	Z	*p*-Value	Rank-Biserial Correlation
Benefits Subscale	Yes	222	186.28	6042.50	−6.745	<0.001	0.466
	No	102	110.74				
Barriers Subscale	Yes	222	142.44	6869.00	−5.695	<0.001	0.393
	No	102	206.16				
EBBS Total	Yes	222	189.33	5366.50	−7.607	<0.001	0.526
	No	102	104.11				

Note. EBBS = Exercise Benefits/Barriers Scale; U = Mann–Whitney U statistic; Z = standardized test statistic; *p*-value = two-tailed probability value. Exercise practice was classified according to the ACSM criterion, defined as planned and structured physical activity of at least moderate intensity, performed for ≥30 min on ≥3 days per week, for at least the last 3 months. Rank-biserial correlations are reported as absolute values; the direction of the differences is indicated by the group mean ranks.

## Data Availability

The data presented in this study are available from the corresponding author upon reasonable request.

## Supplementary Materials

The following supporting information can be downloaded at: https://www.mdpi.com/article/10.3390/healthcare14172849/s1, Table S1: Standardized factor loadings and residual variances for the 43 EBBS items; Table S2: Standardized loadings of the nine first-order factors on the two second-order EBBS factors; Table S3: Correlation and covariance between the second-order latent factors of the EBBS; Table S4: Modification indices for the original 43-item second-order hierarchical CFA model; Table S5: Corrected item-total correlations of the EBBS items; Figure S1: Bland–Altman plot for the EBBS Benefits Subscale; Figure S2: Bland–Altman plot for the EBBS Barriers Subscale.

## Abbreviations

The following abbreviations are used in this manuscript:

ACSM	American College of Sports Medicine
AMOS	Analysis of Moment Structures
CFA	confirmatory factor analysis
CFI	Comparative Fit Index
CI	confidence interval
COSMIN	COnsensus-based Standards for the selection of health Measurement INstruments
df	degrees of freedom
EBBS	Exercise Benefits/Barriers Scale
EMI-2	Exercise Motivation Inventory-2
ICC	intraclass correlation coefficient
IPAQ	International Physical Activity Questionnaire
IPAQ-SF	International Physical Activity Questionnaire—Short Form
LoA	limits of agreement
M1	first assessment
M2	second assessment
MET	metabolic equivalent of task
QR	quick response
RMSEA	Root Mean Square Error of Approximation
SD	standard deviation
SDC	smallest detectable change
SEM	standard error of measurement
SPSS	Statistical Package for the Social Sciences
SRMR	Standardized Root Mean Square Residual
TLI	Tucker–Lewis Index
