# Supplementary material for: Translation, Cross-Cultural Adaptation, and Psychometric Evaluation of the European Portuguese Exercise Benefits/Barriers Scale in Higher Education Students
Source: Healthcare (Basel). 2026 Sep 4;14(17):2849. doi: 10.3390/healthcare14172849 (PMC13565202; doi:10.3390/healthcare14172849)
Supplement: Supplementary file 1 [file healthcare-14-02849-s001.zip › healthcare-4452273-supplementary.pdf]

Supplementary Materials
Translation, Cross-Cultural Adaptation, and Psychometric Evaluation of the European Portuguese Exercise Benefits/Barriers Scale in Higher Education Students

Table S1. Standardized factor loadings and residual variances for the 43 EBBS items.

First-order factor	EBBS item	Standardized factor loading	Residual variance
Psychological Outlook	Item 1	0.729	0.468
Psychological Outlook	Item 2	0.817	0.333
Psychological Outlook	Item 3	0.787	0.381
Time Expenditure	Item 4	0.518	0.732
Preventive Health	Item 5	0.674	0.546
Physical Exertion	Item 6	0.712	0.493
Physical Performance	Item 7	0.656	0.569
Psychological Outlook	Item 8	0.649	0.579
Exercise Milieu	Item 9	0.537	0.712
Psychological Outlook	Item 10	0.806	0.351
Social Interaction	Item 11	0.735	0.460
Exercise Milieu	Item 12	0.583	0.660
Preventive Health	Item 13	0.720	0.482
Exercise Milieu	Item 14	0.530	0.719
Physical Performance	Item 15	0.684	0.533
Exercise Milieu	Item 16	0.546	0.702
Physical Performance	Item 17	0.652	0.575
Physical Performance	Item 18	0.674	0.545
Physical Exertion	Item 19	0.743	0.448
Psychological Outlook	Item 20	0.807	0.349
Family Discouragement	Item 21	0.684	0.532
Physical Performance	Item 22	0.640	0.591
Physical Performance	Item 23	0.561	0.686
Time Expenditure	Item 24	0.766	0.414
Life Enhancement	Item 25	0.750	0.438
Life Enhancement	Item 26	0.698	0.513
Preventive Health	Item 27	0.660	0.564
Exercise Milieu	Item 28	0.504	0.746

First-order factor	EBBS item	Standardized factor loading	Residual variance
Life Enhancement	Item 29	0.643	0.587
Social Interaction	Item 30	0.684	0.531
Physical Performance	Item 31	0.756	0.429
Physical Performance	Item 32	0.762	0.419
Family Discouragement	Item 33	0.714	0.490
Life Enhancement	Item 34	0.751	0.437
Life Enhancement	Item 35	0.706	0.502
Life Enhancement	Item 36	0.720	0.482
Time Expenditure	Item 37	0.860	0.261
Social Interaction	Item 38	0.756	0.429
Social Interaction	Item 39	0.214	0.954
Physical Exertion	Item 40	0.557	0.690
Life Enhancement	Item 41	0.756	0.429
Exercise Milieu	Item 42	0.622	0.614
Physical Performance	Item 43	0.603	0.637

Note. EBBS = Exercise Benefits/Barriers Scale. Standardized factor loadings were obtained from the confirmatory factor analysis of the original second-order hierarchical model. Residual variance was calculated as $1 - R^2$, where R^2 is the squared multiple correlation for each item. All analyses were based on N = 324.

Table S2. Standardized loadings of the nine first-order factors on the two second-order EBBS factors.

Second-order factor	First-order factor	Standardized factor loading
Benefits	Life Enhancement	0.995
Benefits	Physical Performance	0.929
Benefits	Psychological Outlook	0.826
Benefits	Social Interaction	0.762
Benefits	Preventive Health	0.677
Barriers	Exercise Milieu	0.918
Barriers	Time Expenditure	0.632
Barriers	Physical Exertion	0.574
Barriers	Family Discouragement	0.726

Note. EBBS = Exercise Benefits/Barriers Scale. Values represent standardized regression weights from the second-order hierarchical confirmatory factor analysis. All analyses were based on N = 324.

Table S3. Correlation and covariance between the second-order latent factors of the EBBS.

Latent factor 1	Latent factor 2	Covariance	SE	CR	p-value	Correlation
Benefits	Barriers	-0.074	0.016	-4.632	< 0.001	-0.384

Note. EBBS = Exercise Benefits/Barriers Scale; SE = standard error; CR = critical ratio. The covariance is unstandardized, whereas the correlation is standardized. The p-value reported by AMOS as *** corresponds to $p < 0.001$. All analyses were based on $N = 324$.

Table S4. Modification indices for the original 43-item second-order hierarchical CFA model.

Panel A. Covariances.

Parameter 1	Parameter 2	M.I.	Par change
e46	e48	14.276	-0.023
e46	e47	27.658	0.049
e45	e49	13.394	-0.024
e45	e48	16.653	0.016
e40	e52	12.550	0.059
e40	e47	10.493	-0.068
e40	e46	13.414	-0.043
e4	e6	12.420	0.085
e16	e6	10.957	-0.088
e28	e33	14.315	0.085
e12	Benefits	10.821	-0.059
e12	e47	22.048	-0.104
e12	e46	10.684	-0.040
e30	e11	19.623	0.096
e39	Barriers	21.359	0.099
e39	e52	13.449	0.072
e38	e48	10.476	-0.035
e38	e47	10.656	-0.049
e38	e46	25.116	0.046
e38	e40	14.020	-0.077
e38	e12	31.105	-0.122
e20	e46	16.560	-0.026
e3	e46	11.440	0.020
e3	e20	11.407	-0.026
e2	e46	12.727	0.022
e2	e3	66.260	0.060
e1	Barriers	10.684	-0.037
e1	e47	41.331	0.085
e1	e40	12.340	-0.058
e1	e12	29.027	-0.095

Parameter 1	Parameter 2	M.I.	Par change
e1	e11	10.676	0.052
e23	e1	11.243	-0.044
e22	e50	11.402	-0.063
e22	e46	30.043	0.052
e22	e45	28.115	-0.033
e22	e10	20.242	0.060
e22	e23	14.100	0.065
e31	e16	10.056	-0.048
e31	e22	10.261	-0.037
e32	e5	11.011	-0.034
e32	e7	14.339	-0.035
e43	e46	14.424	-0.029
e43	e3	10.705	-0.030
e18	e48	50.033	0.055
e18	e47	16.617	-0.048
e18	e5	37.176	0.059
e18	e32	11.645	-0.030
e17	e21	12.222	-0.057
e17	e7	25.700	0.050
e17	e18	14.720	0.036
e15	e48	10.703	0.026
e15	e28	11.017	-0.045
e15	e18	41.776	0.054
e36	e35	56.227	0.092
e41	e46	11.865	-0.022
e41	e45	27.437	0.022
e41	e31	15.017	0.030
e41	e43	13.318	0.035
e34	e36	10.336	0.036
e29	e50	19.479	-0.090
e29	e22	21.091	0.090
e26	e46	10.462	0.026

Parameter 1	Parameter 2	M.I.	Par change
e25	e46	24.306	0.034
e25	e23	13.887	0.047
e25	e22	40.411	0.083
e25	e35	24.921	-0.054
e25	e26	10.553	0.035

Panel B. Regression weights.

Parameter 1	Parameter 2	M.I.	Par change
TimeExpenditure	SocialInteraction	12.146	0.130
EBBS33	EBBS28	13.840	0.187
EBBS6	EBBS16	14.050	-0.156
EBBS40	Barriers	14.623	0.391
EBBS40	Benefits	10.634	-0.291
EBBS40	TimeExpenditure	10.996	0.351
EBBS40	ExerciseMilieu	16.918	0.379
EBBS40	SocialInteraction	19.103	-0.293
EBBS40	PsychologicalOutlook	19.952	-0.409
EBBS40	LifeEnhancement	10.292	-0.284
EBBS40	EBBS16	17.618	0.189
EBBS40	EBBS42	12.360	0.166
EBBS40	EBBS12	17.714	0.191
EBBS40	EBBS38	26.355	-0.274
EBBS40	EBBS10	14.765	-0.226
EBBS40	EBBS20	12.234	-0.218
EBBS40	EBBS3	10.214	-0.230
EBBS40	EBBS2	19.584	-0.284
EBBS40	EBBS1	29.198	-0.345
EBBS24	EBBS30	13.147	0.164
EBBS4	EBBS6	15.977	0.192
EBBS12	SocialInteraction	24.561	-0.352
EBBS12	PsychologicalOutlook	16.661	-0.395

Parameter 1	Parameter 2	M.I.	Par change
EBBS12	EBBS30	13.514	-0.194
EBBS12	EBBS38	43.325	-0.372
EBBS12	EBBS3	16.156	-0.306
EBBS12	EBBS2	14.819	-0.261
EBBS12	EBBS1	40.385	-0.429
EBBS5	EBBS18	16.574	0.202
EBBS39	Barriers	17.585	0.507
EBBS39	ExerciseMilieu	19.949	0.485
EBBS39	EBBS40	10.492	0.187
EBBS39	EBBS16	12.459	0.187
EBBS39	EBBS28	11.295	0.215
EBBS39	EBBS42	13.146	0.202
EBBS39	EBBS12	13.983	0.200
EBBS38	Barriers	14.276	-0.302
EBBS38	PhysicalExertion	10.818	-0.204
EBBS38	ExerciseMilieu	14.749	-0.276
EBBS38	PsychologicalOutlook	18.962	0.311
EBBS38	EBBS40	20.425	-0.173
EBBS38	EBBS12	39.263	-0.222
EBBS38	EBBS8	12.317	0.177
EBBS38	EBBS10	15.460	0.181
EBBS38	EBBS20	17.266	0.203
EBBS38	EBBS3	13.408	0.206
EBBS38	EBBS2	12.443	0.177
EBBS38	EBBS1	19.213	0.219
EBBS8	EBBS43	13.995	0.173
EBBS8	EBBS17	10.362	0.151
EBBS10	EBBS22	11.792	0.112
EBBS20	EBBS31	10.852	0.138
EBBS20	EBBS32	10.595	0.121
EBBS20	EBBS18	12.420	0.153
EBBS3	EBBS2	18.941	0.143

Parameter 1	Parameter 2	M.I.	Par change
EBBS3	EBBS43	14.465	-0.133
EBBS2	EBBS3	22.476	0.188
EBBS2	EBBS17	13.983	-0.142
EBBS2	EBBS41	10.253	-0.123
EBBS1	SocialInteraction	11.127	0.140
EBBS1	EBBS40	16.194	-0.124
EBBS1	EBBS12	32.855	-0.163
EBBS1	EBBS11	17.571	0.117
EBBS1	EBBS30	14.467	0.119
EBBS1	EBBS38	12.008	0.116
EBBS22	PsychologicalOutlook	14.360	0.285
EBBS22	EBBS6	10.419	-0.130
EBBS22	EBBS10	28.856	0.260
EBBS22	EBBS20	10.721	0.168
EBBS22	EBBS3	10.172	0.189
EBBS22	EBBS1	10.343	0.169
EBBS22	EBBS29	18.662	0.172
EBBS22	EBBS25	25.535	0.266
EBBS7	EBBS17	13.960	0.152
EBBS43	EBBS3	10.169	-0.153
EBBS18	PreventiveHealth	15.778	0.262
EBBS18	EBBS13	15.987	0.134
EBBS18	EBBS5	41.132	0.251
EBBS18	EBBS15	20.782	0.191
EBBS17	EBBS7	13.785	0.172
EBBS15	EBBS18	21.354	0.197
EBBS35	EBBS36	25.244	0.205
EBBS35	EBBS25	10.015	-0.139
EBBS36	FamilyDiscouragement	10.227	0.179
EBBS36	EBBS35	26.454	0.207
EBBS41	EBBS31	11.277	0.135
EBBS41	EBBS43	13.035	0.135

Parameter 1	Parameter 2	M.I.	Par change
EBBS29	PhysicalExertion	12.160	-0.249
EBBS29	EBBS19	12.814	-0.166
EBBS29	EBBS6	10.055	-0.140
EBBS29	EBBS22	10.460	0.156
EBBS25	EBBS22	22.189	0.152
EBBS25	EBBS35	11.740	-0.123

Note. CFA = confirmatory factor analysis; EBBS = Exercise Benefits/Barriers Scale; M.I. = modification index; Par change = expected parameter change. Panel A reports covariances and Panel B reports regression weights suggested by IBM SPSS Amos for fixed parameters in the original second-order hierarchical model. Only modification indices with M.I. ≥ 10.000 are reported to highlight the most substantial potential sources of model misfit and maintain interpretability. These indices are diagnostic outputs only; none of the suggested parameters were added to or estimated in the tested model. Analyses were based on N = 324.

Table S5. Corrected item–total correlations of the EBBS items.

EBBS Item	Benefits Subscale	Barriers Subscale	EBBS Total
Item 1	0.623	—	0.640
Item 2	0.631	—	0.613
Item 3	0.606	—	0.572
Item 4	—	0.438	0.244
Item 5	0.416	—	0.378
Item 6	—	0.358	0.336
Item 7	0.590	—	0.541
Item 8	0.619	—	0.561
Item 9	—	0.465	0.259
Item 10	0.704	—	0.675
Item 11	0.549	—	0.538
Item 12	—	0.492	0.468
Item 13	0.464	—	0.403
Item 14	—	0.447	0.346
Item 15	0.613	—	0.548
Item 16	—	0.444	0.315
Item 17	0.568	—	0.514
Item 18	0.601	—	0.514
Item 19	—	0.430	0.324
Item 20	0.748	—	0.718
Item 21	—	0.403	0.312
Item 22	0.656	—	0.612
Item 23	0.509	—	0.458
Item 24	—	0.502	0.246
Item 25	0.734	—	0.673
Item 26	0.665	—	0.606
Item 27	0.521	—	0.456
Item 28	—	0.426	0.277
Item 29	0.624	—	0.596
Item 30	0.483	—	0.452
Item 31	0.675	—	0.624
Item 32	0.721	—	0.678

EBBS Item	Benefits Subscale	Barriers Subscale	EBBS Total
Item 33	—	0.487	0.393
Item 34	0.705	—	0.630
Item 35	0.654	—	0.571
Item 36	0.671	—	0.583
Item 37	—	0.560	0.303
Item 38	0.658	—	0.667
Item 39	0.175	—	0.065
Item 40	—	0.491	0.448
Item 41	0.708	—	0.666
Item 42	—	0.522	0.353
Item 43	0.545	—	0.472

Note. EBBS = Exercise Benefits/Barriers Scale. A dash (-) indicates that the item does not belong to the corresponding subscale. Corrected item–total correlations were calculated separately for the Benefits Subscale, Barriers Subscale, and EBBS Total score. For the EBBS Total score, barrier items were reverse-coded. All analyses were based on N = 324, with no missing data.

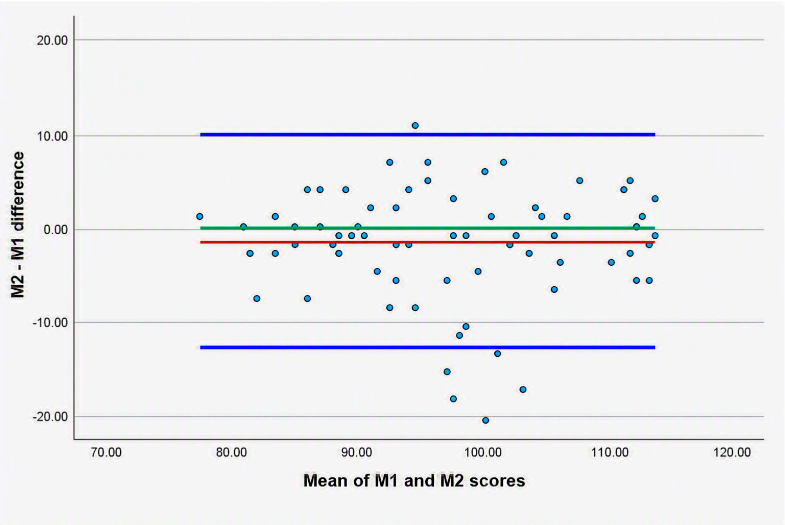

Figure S1. Bland–Altman plot for the EBBS Benefits Subscale. The green line represents zero difference between M2 and M1, the red line represents the mean difference (bias), and the blue lines represent the 95% limits of agreement.

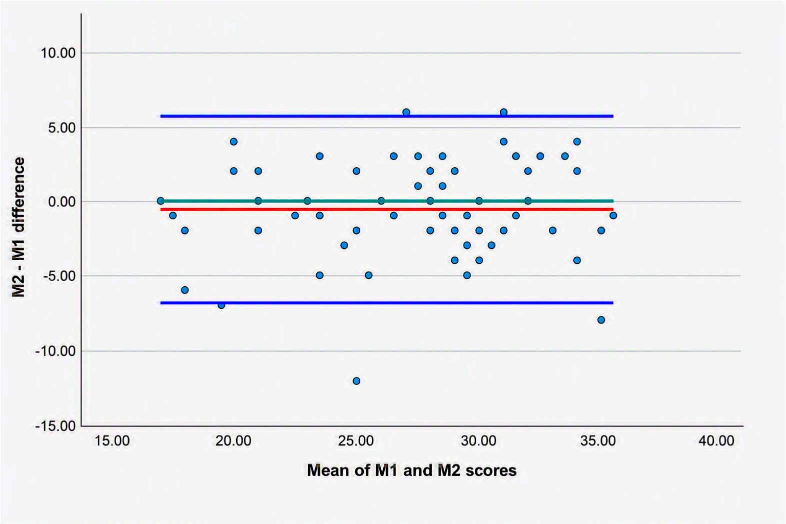

Figure S2. Bland–Altman plot for the EBBS Barriers Subscale. The green line represents zero difference between M2 and M1, the red line represents the mean difference (bias), and the blue lines represent the 95% limits of agreement.
